# Undergraduate Nursing Students’ Experiences of Conducting Clinical Research Projects in Their Bachelor Theses – a Qualitative Study

**DOI:** 10.1177/23779608221094537

**Published:** 2022-04-20

**Authors:** Kjersti Grønning, Guro Karlsholm, Beate André

**Affiliations:** 1 Department of Public Health and Nursing, 8018 Norwegian University of Science and Technology, 7491 Trondheim, Norway; 2 Department of Research, 60500 Nord-Trøndelag Hospital Trust, Postboks 333, 7601 Levanger, Norway

**Keywords:** Evidence-based nursing, undergraduate nursing students, nursing education, bachelor theses, research

## Abstract

**Introduction:**

The aim of educational institutions in the field of nursing is to educate nurses with the competences to provide high-quality care to their patients, meaning that students need to learn about evidence-based practice and how to translate the knowledge, skills, competency, beliefs, attitudes, and behaviours into daily practice. The bachelor thesis is the ultimate test for undergraduate nursing students to present what they have learned.

**Objective:**

The aim of this study was to explore undergraduate nursing students’ experiences of conducting clinical projects in their bachelor theses.

**Methods:**

We used qualitative focus-group interviews to gain a deeper understanding of the students’ experiences of conducting clinical projects in their bachelor theses as an approach to learn about evidence-based practice. The analyses focused on meaning according to Brinkmann and Kvale.

**Results:**

Eighteen out of 22 eligible students who had chosen to participate in ongoing clinical research projects and write an academic paper as their bachelor thesis were included in this study. The students were all females and divided into three focus groups. The clinical projects were conducted in a public hospital, a private hospital, nursing homes, or within home-care nursing. The analyses showed that conducting clinical projects in the bachelor thesis provided the students with important knowledge for providing evidence-based care and it. motivated them to want to initiate future clinical projects as nurses., They got valuable hands-on experience for how to conduct research, and made the students aware of the importance of keeping themselves updated on the latest knowledge.

**Conclusion:**

Conducting clinical research projects on a bachelor level provide undergraduate nursing students with important knowledge of how to provide evidence-based nursing care to their patients. Learning how to conduct clinical research projects is also important for motivating future nurses to initiate research aiming to improve clinical nursing practice.

## Introduction

One essential task for educational institutions in the field of nursing is to provide a curriculum that educates nurses with the competence to meet real-world demands, where a more structured and holistic way of teaching and assessing evidence-based practice (EBP) (Kumah et al., 2021) and creating a culture that uses evidence to update clinical practice is needed (Kalb et al., 2015). EBP is defined as “*conscientious, explicit, and judicious use of current best evidence in making decisions about the care of individual patients….integrating the best available external clinical evidence from systematic research”* (Sackett et al., 1996). Undergraduate nursing students must learn to translate knowledge, skills, competency, beliefs, attitudes, and behaviours into daily practice and improve clinical outcomes (Patelarou et al., 2020).

The Sicily Statement puts forward a five-step model on how to teach and conduct EBP: (I) ask a clinical question; (II) collect the most relevant evidence; (III) critically appraise the evidence; (IV) integrate the evidence with one's clinical expertise, patient preferences, and values to make a practice decision; and (V) evaluate the change or outcome (Burns & Foley, 2005). There are a range of teaching strategies about EBP in undergraduate nursing education that make the students more confident in accurately interpreting the literature and research (Horntvedt et al., 2018; Wakibi et al., 2021). Teaching students about evidence-based nursing is important for the students’ learning process of becoming good critical thinkers and learning the skills of processing problems, recognizing assumptions, and deducing, interpreting, and evaluating arguments (Cui et al., 2018). Various educational interventions are shown to be effective in providing nurses with sufficient competences to apply EBP in clinical practice (Häggman-Laitila et al., 2016), develop a better assessment of the reliability/validity of information, and become more comfortable with the concepts of EBP (Kyriakoulis et al., 2016). Research courses and workshops are the most frequently used teaching methods (Larsen et al., 2019). Nevertheless, since approaches that engage students in clinical EBP projects are reducing the barriers of EBP, educational institutions must look at nursing curriculum models that support the integration of EBP competencies beyond the classroom (Fiset et al., 2017). Collaboration with clinical practice is an effective way to teach EBP (Horntvedt et al., 2018), where all nurses, including the staff in clinical settings, have a role to play in the students’ learning of EBP (Wakibi et al., 2021).

The curricula for nursing undergrad students must include learning goals for attitudes, knowledge, skills, and practice, with a special focus on the implementation and use of new evidence to improve patient care. Effective learning of EBP for undergraduate students is complex, and cognitive and behavioural components are necessary for success (Ramis et al., 2019). In current nursing education in Norway, undergraduate students learn about the research process, but not in relation to EBP or clinical practice (André et al., 2016; Hole et al., 2016). EBP has the potential to change clinical practice and increase care quality by making the nursing care process more individually tailored to patients’ situations (Yost et al., 2015). The Institute of Medicine's Quality Chasm series suggests that EBP is one of ﬁve core competencies for professional healthcare curricula (Ubbink et al., 2013). Evidence and knowledge in nursing develop from several sources, and different kinds of knowledge are necessary to meet the wide range of ill and needy persons (Martinsen & Eriksson, 2009).

One way to expand EBP in education and integrate scientific thinking in clinical practice is to use clinical practice as an arena for undergraduate nursing students to seek research knowledge for solving specific clinical problems (Moch et al., 2015). Furthermore, to apply critical analytical assessment of research-based knowledge in clinical situations, students need exercise in how to use such knowledge, e.g., by participating in ongoing clinical research projects under supervision (André et al., 2016).

The writing of a bachelor thesis plays a central role within higher education and can be described as the ultimate test for undergraduate students in nursing. Students learn alternative ways of thinking through the process of working on a bachelor thesis (Halabi & Hamdan-Mansour, 2012). Student knowledge about important issues regarding nursing care both increases and deepens by writing a bachelor thesis (Lundgren & Halvarsson, 2009). Students have also stated that by working on their bachelor thesis they expected to gain valuable knowledge needed for their professional practice in the health care services (Henttonen et al., 2021). Working on a bachelor thesis is a complex process and includes learning activities that consist of more than just reading the literature and attending lectures and seminars (Lundgren & Halvarsson, 2009; Mattila et al., 2005; Mattsson, 2016). It provides the students with an opportunity to independently work on a complex assignment, formulate a topic, select relevant literature, and process the data (André et al., 2016; Roca et al., 2018). A pilot-study found that students who conducted clinical projects as part of their bachelor thesis reported that their participation influenced their attitudes towards EBP (André et al., 2016). The students experienced an increased understanding of the importance of critical thinking and realized how important it was to use research to solve problems and increase knowledge related to phenomena in nursing practice (André et al., 2016).

Targeted qualitative research is needed to explore how to teach evidence-based practice in nursing education (Horntvedt et al., 2018). Hence, the aim of this study is to explore undergraduate nursing students’ experiences of conducting clinical projects in their bachelor theses.

## Methods

### Design

The setting for this qualitative focus-group study was the Faculty of Medicine, Department of Public Health and Nursing at the Norwegian University of Science and Technology (NTNU). Around 250 undergraduate nursing students are educated at NTNU each year. The programme is a full-time course with a total of 180 ECTS credits over three years (six semesters) and is equally divided between theoretical and clinical studies. The programme leads to a Bachelor in Nursing degree that qualifies for authorisation as a registered nurse in Norway. All students write their bachelor thesis as a final exam (15 ECTS). The students can choose between conducting a literature review as the basis for their bachelor thesis or participate in ongoing clinical research projects and write an academic paper (NTNU, 2019). Information on the pedagogical scheme for 1) conducting a literature review, or 2) participating in ongoing clinical research projects are presented in the introductory lecture to the course and on the students’ digital learning platform. The students apply for clinical research projects and indicate which projects they prefer to participate in, and by the end of the fifth semester the students are assigned to a project. All students must formulate a research question, attend theoretical lectures and bachelor seminars, and have mandatory individual supervision.

### Sample

An invitation e-mail was sent to all the students (N  =  22) assigned to the pedagogical scheme of participating in ongoing clinical research projects while writing their bachelor thesis. The e-mail was sent at the beginning of the sixth semester and included information about the purpose of the study, topics for the focus-group interview, and the time and place for the interviews.

### Data Collection

The first and last authors conducted three qualitative focus-group interviews (Brinkmann & Kvale, 2015). A thematic interview guide (Table 1) with open-ended main questions, followed by probing sub-questions (e.g., ‘could you give an example of’, ‘is there anything else you would like to add’) was used. The interviews lasted approximately 45 min and were carried out in connection with lectures. All interviews were digitally recorded and transcribed verbatim by a professional transcriber service.

**Table 1.** Interview Guide.

Main themes	Questions
The Bachelor theses	Why did you choose to conduct clinical projects in your bachelor theses – please explain? Could you tell a bit how you experienced to conduct your clinical projects? ○ Preparation, collaboration, guidance ○ What did you learn? How has conducting clinical projects in your bachelor theses influenced your nursing? – please explain why/why not
Evidence based nursing practice	How would you describe / what is evidence-based nursing practice? Have you learned about evidence-based nursing practice in any of your classes? ○ Please tell - give examples In your opinion – what do you think is the best way to learn evidence-based nursing practice ○ Please elaborate

### Data Analysis

The analyses focused on meaning as described in Brinkmann and Kvale (Brinkmann & Kvale, 2015) to explore how undergraduate nursing students experienced conducting clinical projects in their bachelor theses. The first author inductively read and coded all interviews. The research team had several physical and digital meetings to discuss the content and labels of the codes, during which different interpretations were developed until consensus of interpretation was reached. The final analytical themes were agreed upon by comparing (finding similarities) and contrasting (searching for negative cases) codes. NVivo 11.0 (QSR, 2007), a data management program, was used as a tool to systematise and code the transcriptions.

### Ethical Considerations

Participation in the study was voluntary. All students received oral and written information about the purpose of the study and gave written consent to participate. The Norwegian Centre for Research Data approved the study.

## Results

Out of 22 eligible nursing students (21 females and 1 male), 18 responded to the invitation. The students (ages ranging from 22–45 years old) were divided into three focus groups. All interviewed students were females, and each focus group consisted of six students. The students participated in nine different clinical projects in a public hospital, private hospital, nursing home, or through home-care nursing, and the topics were developed in collaboration between the university and clinical practice. The topics for the project were all related to clinical nursing practice in medical, surgical, or palliative units in the hospital, or in nursing homes and home care.

The analyses showed that by conducting clinical projects in their bachelor thesis the students gained important knowledge for providing evidence-based care. This was also motivating for initiating clinical projects in the future and gave valuable hands-on experience for how to carry out research; students also became aware of the importance of keeping themselves updated on the latest knowledge. Through the process of conducting clinical projects the students also reflected on nursing and clinical practice, what constitutes evidence and truth, and that role models and unit culture have a big impact when someone suggests change. An overview of the final themes and subthemes are illustrated in Table 2.

**Table 2.** Final Themes and Sub-Themes.

Final theme	Sub-themes
Important knowledge for providing evidence-based care	Motivating for initiating future projects as nurses
	Hands-on practice of EBP
	Necessity of professional updating
Critical reflection about nursing and clinical practice	What is evidence and truth
	Role models and culture
	Initiate changes

The analyses also showed that the students chose to conduct clinical projects in their bachelor theses because they wanted to try something different, or they had a special interest in the topics that were offered. Some students had been advised by former students to choose this alternative for their bachelor thesis, while others said they chose clinical projects because they had positive experiences with clinical placements at the units offering the projects. The results are further elaborated with quotes from the students (focus group number and informant) below.

### Important Knowledge for Providing Evidence-Based Care

The students experienced their clinical projects as important for improving clinical practice. Conducting clinical projects motivated and gave the students valuable hands-on practice for how to initiate clinical projects in the future. The process of conducting clinical projects also gave the students important insights into EBP in practice, as well as in the importance of professional updating.

Their research questions were created based on a topic the clinical units wanted explored. They said the hands-on experience of conducting their own studies and carrying out the research process, step-by-step, was useful knowledge for them in their future work-life as registered nurses. The hands-on experience of doing research, sharing their results with fellow students and nurses, and getting constructive feedback were exciting and made them proud. The students said it was fun and stimulating to dig deeper into an area of particular interest. By having their own project, the students gained special ownership of their topic, data, and final bachelor thesis. By getting hands-on practice on how to collect data, the students also thought it would be easier to conduct new projects in the future.

*“You learn a lot by conducting your own data collection. You get new ideas for doing more research, or participate in clinical projects when you start to work as a nurse.”*

(Informant E, group 1)

*“For me, it was important to collect my own data; it was easier to implement the theory into clinical practice because you had done it yourself.”*

(Informant B, group 2)

The students further explained that conducting clinical projects could be a good way to learn about evidence-based nursing. Some students said they contributed to EBP by conducting clinical projects. When asked if they had heard and/or learned about the concept of evidence-based practice earlier in their education, they remembered another theoretical course that focused on it and that a previous exam had questions about evidence-based nursing. When the students were asked how they understood the concept of evidence-based nursing, two expressed it like this:

“*I think evidence-based nursing is about, for instance, adapting a procedure to how the patient wants it done, or in changing the mattress (even though you know that this is the best type), if the patient tells you that he can’t sleep on a that mattress.”*

(Informant A, group 3)

“*I think it is about using research and experiences….and I guess…. it was something about the patient's…user involvement. That it is important to offer the best care.”*

(Informant A, group 2)

The students further said that by doing clinical projects and producing valuable knowledge for clinical practice, they had become extra aware of the importance of keeping themselves updated while working as registered nurses. They also realised that professional updating on a busy clinical day with a heavy workload could be challenging. Some were a bit concerned they would not have enough time to keep up with recent research when they started to work as nurses. When discussing how to keep up with recent research, the students had different experiences from clinical practice on how the hospital units, nursing homes, and home care nursing staff prioritised professional updating and innovation. All students could tell they had taken part in different kinds of professional meetings or internal educational programs during their clinical practice. One student stated that her clinical supervisors had encouraged her to read all kinds of literature and procedures to stay updated on recent knowledge that was relevant for providing high quality care to the patients in that unit. Others experienced that the nurses from another unit were not updated on the latest procedures, particularly on how to heal wounds. Their good and bad experiences regarding how to stay updated on recent knowledge made the students reflect on the importance of having routines for professional updating. Two students expressed it like this:

“*In psychiatry there was a lot of internal teaching about communication and such, much more than in the somatic hospital, almost every day.”*

(Informant A, group 2)

“*If your colleagues at work are like “no* *…”, then it is hard to carry on by yourself…. You have to collaborate and work in teams to stay professionally updated.”*

(Informant D, group 2)

### Critical Reflection About Nursing and Clinical Practice

During the process of conducting their clinical projects the students reflected on what is really evidence and truth. They observed nurses who were good role models, and cultures that were supportive when someone initiated changes based on new knowledge. While preparing their projects, collecting data, and analysing data, the students became conscious about not taking reproduced theory for granted. They questioned why the theory existed, the message of the theory, and the theory's relevance in the setting. They also reflected on their position as students, and that they brought the newest theory or knowledge about nursing into clinical nursing practice. Some students also said that when they were reading scientific papers, they started to question the research questions in the article, the validity of the data, how the data was collected, who collected the data, and in which settings the data were collected.

*“We have been told to be critical, but now I really understand how to be critical, not only with the results but also with the preparations in the study.”*

(Informant B, group 1)

The students further said they had become conscious about how difficult it was to formulate research questions, and anxious about whether or not their questionnaire was sufficient to capture the phenomena they wanted to study. By analysing data, the students said they became critical of their own results and if the results were just a coincidence or “true”. This learning experience opened a new perspective of how to move forward with their results and how their results could contribute to areas for improvement. The students found it interesting that their in-depth investigation of a chosen phenomenon created new knowledge that could initiate changes and improve clinical practice. Two of the students described it like this:

*“We are able to contribute to changes as a result of our project, and it will influence our work as future nurses.”*

(Informant C, group 3)

*“We have actually contributed to possible changes and others have become interested in our project.”*

(Informant A, group 1)

When discussing areas for improvement, several students experienced that some nurses were sceptical of changes and preferred that things remained as they were. Those who had experienced “old-fashioned” nurses were clear that they wanted to be different as nurses. The students stated they experienced how the culture in different clinical units influenced how easy or difficult it was to implement new knowledge.

“*It is easy when you start working to quickly adopt the culture at the workplace. You may have learned one thing at school, but everyone at the clinic does it a different way. Perhaps it is easier said than done.”*

(Informant B, group 2)

The students also talked about the challenging experience of searching for research to use in their thesis. When asked if they had observed nursing research during their clinical studies, almost all students said that research was not discussed. During the process of searching for research papers and conducting their own studies, the students thought there was too little research on nursing and nursing interventions. Most of the projects they had heard about during their clinical placements were conducted by medical doctors, and the nurses only participated as assistants or study nurses. This discovery made the students question why it was like this. Some students argued that the reason was partly because the nurses did not have time to do research, or that clinical nurses traditionally did not conduct their own research. Others said that research was not prioritized among nurses, or that nurses did not have the same opportunities or traditions as medical doctors.

*“The university hospital has a lot of resources, many professors and chief physicians who have funds for research.”*

(Informant C, group 2)

*“It is much more common for doctors to have projects than nurses.”*

(Informant A, group 2)

*“Research is also a part of studying to become a nurse. There are some who actually study nursing because they want to do research. All nurses are not expected to work clinically with patients and carry out procedures and such. Therefore, there must be a greater understanding among nursing teachers about that.”*

(Informant C, group 3)

One student explained that she had a clinical supervisor who was a co-worker in an ongoing clinical research project. This nurse had reserved time to participate in the project. The students further discussed that they had become more aware of the importance of research and developing new knowledge about nursing while they conducted their own projects. They were also very proud of their projects because the units valued their results. Several experienced the clinical nurses as curious and enthusiastic about the results’ influence on clinical nursing practice, while others experienced the nurses as sceptical towards their projects. Some students stated they received positive feedback from patients when they collected data on nurses’ hand hygiene in their project. Even though the students were proud of producing results, some discovered the results were not “good news” about clinical practice. They were a bit concerned about presenting the results in the clinic but were confident that the findings were important for improving nursing practice. One student expressed it like this:

*“This project can actually affect the unit, a change can happen as a result from this thesis, and it will impact us as nurses.”*

(Informant C, group 3)

The students also reflected on what sort of competence they gained from doing their own clinical projects, even though their research projects were on a bachelor level. They expressed that collaborating with other students was very valuable for collecting and analysing data, and interpretating the findings. Another important collaborator was the clinical field, and the unit, nursing home, or home care nursing facilitated data-collection.

## Discussion

This study shows that undergraduate nursing students gained important knowledge about EBP while conducting clinical projects as part of their bachelor theses. The students learned how the research process is carried out in practice, which is shown to be essential for understanding how evidence-based knowledge is developed and is fundamental for the quality of provided nursing care (Patelarou et al., 2020; Ryan, 2016). Incorporation of EBP into the curriculum for undergraduate nursing students is therefore important, and it is important to start training in EBP at the undergraduate level (Patelarou et al., 2020).

### Clinical Projects in the Bachelor Thesis – A New Approach to Learn About EBP

In this study the results showed that conducting clinical projects as part of the undergraduate nursing students’ work with their bachelor theses is one pedagogical way for students to learn about EBP, and to translate their knowledge, skills, competency, beliefs, attitudes, and behaviours into daily practice (Patelarou et al., 2020). This study also found that the students’ engagements in their research projects positively affected the clinical units’ research culture, emphasising the importance of including evidence in clinical nursing practice (Kalb et al., 2015). Despite knowing that teaching EBP to undergraduate nursing students is important (Ryan, 2016), it is shown that some faculty personnel at universities are not familiar with EBP or the need to use evidence in their teaching (Kalb et al., 2015). Our findings indicate that knowledge and understanding of research in nursing education among nursing teachers must be addressed. The students in this study stated that some undergraduate nursing students study nursing because they want to become researchers later on, which is something the teachers need to keep in mind when teaching and supervising students.

Collaboration with clinical practice is shown to be a good strategy for teaching students about EBP (Larsen et al., 2019); for instance, by letting students conduct clinical projects as part of their bachelor theses, as this study shows. Our findings illustrate that this approach is one way for students to learn how the knowledge base for EBP is created and expanded. Larsen and co-workers (Larsen et al., 2019) also point out that both the combination of classrooms and clinical practice settings, and in clinical practice settings alone, are effective ways to learn EBP. However, increased collaboration with clinical practice needs to be strengthened since EBP teaching strategies including clinical activities in nursing students are less prioritised (Horntvedt et al., 2018). Furthermore, EBP teaching methods are found to be successful learning approaches for becoming good critical thinkers (Cui et al., 2018). EBP teaching methods teach students how to process problems and recognize the assumption, deduction, interpretation, and evaluation of arguments (Cui et al., 2018). The pedagogical teaching strategy in this study requires that the students formulate a research question, collect data, analyse data, and interpret the findings in relation to the existing literature. Their clinical experiences go behind the range of pedagogical strategies that only teach undergraduate nursing students how to interpret the literature in order to provide patients with evidence-based care (Cui et al., 2018; Horntvedt et al., 2018; Häggman-Laitila et al., 2016; Wakibi et al., 2021). Conducting research on a bachelor level represents a different approach to engaging students in learning about EBP that supports the integration of EBP competencies beyond the classroom (Fiset et al., 2017; Horntvedt et al., 2018). Our findings provide knowledge about a successful pedagogical approach for how to teach undergrade students about EBP that contains of more than participating in research courses and workshops (Larsen et al., 2019). Our pedagogical approach also includes carrying out research on a bachelor level. Undergraduate nursing students who conduct research on a bachelor level learn EBP in a context where both nurse educators and clinical staff play a key role in students’ learning of EBP (Wakibi et al., 2021).

Furthermore, this study shows that the students got important hands-on experience for how to carry out clinical research by doing small projects as part of their bachelor theses. This was a valuable experience that made the students more aware of their responsibility of keeping themselves updated on the latest knowledge as nurses. This awareness confirms the findings from other studies (Cui et al., 2018; Halabi & Hamdan-Mansour, 2012), showing that students learn alternative ways of thinking through the process of working with their bachelor theses and gain an increased and deepened knowledge about nursing care (Henttonen et al., 2021; Lundgren & Halvarsson, 2009).

Our study also found that the students reflected on what ‘evidence’ is through the process of conducting clinical projects, which confirms findings from other studies (André et al., 2016; Roca et al., 2018) that highlight the importance of providing students with an opportunity to independently work on complex assignments that go beyond searching for relevant literature and attending lectures (Lundgren & Halvarsson, 2009; Mattila et al., 2005; Mattsson, 2016).

In this study the results also showed that by working on clinical projects for a bachelor thesis, the students became conscious about the importance of initiating changes if clinical nursing practice was not based on the newest recommendations or guidelines. Other studies have found that to increase the quality of nursing it is important for undergraduate nursing students to expand the use of EBP in clinical practice (André et al., 2016; Florin et al., 2012). The students must learn how to evaluate research findings, reflect on current practice, and incorporate EBP knowledge into clinical practice in order to continue such activities as registered nurses. If undergraduate nursing students incorporate a positive attitude towards nursing research during their education, they will likely appreciate the value of research in their clinical practice as nurses (Moch et al., 2015).

The results of this study showed that the students learned that the clinical units had different cultures concerning research, which may be due to lack of education or training on how to improve EBP (Cullen et al., 2011; Moch et al., 2015). One way to prevent barriers toward EBP is to establish partnerships and collaborate in clinical projects that aim to improve nursing practice. Adopting evidence-based practices within complex health care organizations is challenging, and educators have a responsibility to increase emphasis on skills that nurses need to possess to oversee evidence-based practice initiatives (Cullen et al., 2011). However, more research is needed to assess effective ways of teaching EBP to both nursing students (Patelarou et al., 2020) and nurses.

### Strengths and Limitations

One of the strengths in this study is the chosen methodology used to gain in-depth knowledge about the students’ experiences of conducting clinical projects in their bachelor theses. By conducting qualitative focus-group interviews (Brinkmann & Kvale, 2015) and asking open-ended questions, the students were invited to discuss their experiences with the other students and illustrate their experiences with examples. The focus-group discussions generated an understanding inside the theme as experienced by the students. Using a semi-structured method, the interviews offered space for the students to deepen their viewpoints and provide data on how they experienced conducting clinical projects. Another strength is the composition of the focus groups: some students knew each other well because they collaborated when they conducted their projects, while other students were less known to each other. The students’ familiarity with each other might have contributed to a more relaxed and free-speaking session. Another strength is that the analysis and writing of the paper were conducted by a group of researchers. This helped ensure the reliability of the findings. Dependability and confirmability are major elements in identifying the implications of this study, and much work was committed to exploring these topics (Miles et al., 2020). Interpretations of the data were frequently discussed among the authors to confirm the dependability of the results. There are also s few limitations in this study. One limitation is that no male students participated in the focus-group interviews. Another limitation is the small sample size. These limitations may weaken the transferability of the findings to other settings and to other undergraduate nursing students.

### Implications for Clinical Practice

The students conducted their projects in collaboration with nurses in different units in the hospital, nursing homes, or in home care nursing. The topics varied a great deal - from nurses’ roles in relation to patient safety, proper drug use, e-health consultations, hygiene, the use of surgical check-lists, and occupational health, to seniors’ perspectives of using apps for preventing loneliness among older adults. The variations in themes and settings for the projects show that the findings have implications for clinical practice in both specialised (hospitals) and primary health care (nursing homes and home-care nursing) since the nursing students gained knowledge on how to provide evidence-based nursing care across settings in the health care services.

## Conclusion

This study shows that conducting clinical research projects on a bachelor level provides undergraduate nursing students with important knowledge on how to provide evidence-based nursing care. The projects provide the students with valuable hands-on experience on how to carry out research and makes them aware of the importance of keeping themselves updated on the latest knowledge. Conducting clinical research projects is also important in motivating the students, as future nurses, to initiate projects that aim to improve clinical nursing practice and to reflect on actual evidence and truth in different settings.
